# MIR337-3p Enhances Mycobacterial Pathogenicity Involving TLR4/MYD88 and STAT3 Signals, Impairing VDR Antimicrobial Response and Fast-Acting Immunity

**DOI:** 10.3389/fimmu.2021.739219

**Published:** 2021-11-29

**Authors:** Shanshan Liang, Guixian Huang, Tian Wu, Ying Peng, Xi Liu, Xuejiao Ji, Wei Sha, Feifei Wang, Ling Shen, Hongbo Shen

**Affiliations:** ^1^ Clinic and Research Center of Tuberculosis, Shanghai Institute of Infectious Disease and Biosecurity, Shanghai Key Laboratory of Tuberculosis, Shanghai Pulmonary Hospital, Institute for Advanced Study, Tongji University School of Medicine, Shanghai, China; ^2^ Key Laboratory of Medical Molecular Virology (Ministry of Education of the people's Republic of China (MOE)/National Health Commission of the people's Republic of China (NHC)/Chinese Academy of Medical Sciences (CAMS)), Department of Medical Microbiology and Parasitology, School of Basic Medical Sciences, Shanghai Medical College, Fudan University, Shanghai, China; ^3^ Department of Microbiology & Immunology and Center for Primate Biomedical Research, University of Illinois College of Medicine, Chicago, IL, United States

**Keywords:** *Mycobacterium tuberculosis*, miR337-3p, vitamin D3, TLR4, STAT3, CYP27B1

## Abstract

Active form of vitamin D (VitD) enhances human innate immunity against *Mycobacterium tuberculosis* (*Mtb*) infection. Our previous studies showed that MIR337-3p was highly expressed in lymphocytes of tuberculosis (TB) patients. Here, we identified the mechanism of MIR337-3p in the regulation of fast-acting anti-TB immunity by inhibiting VitD-dependent antimicrobial response pathways. While high-level MIR337-3p expression was induced by mycobacterial infection in cellular models and mice, TB patients exhibited significantly increased MIR337-3p in CD14^+^ monocytes/macrophages, innate-like Vγ2^+^ T cells, and CD8^+^ lymphocytes containing natural killer (NK)/innate lymphoid cells. MIR337-3p promoted the mycobacterial entry/infection and replication/growth in host target cells: macrophages and lung epithelial cells. Such MIR337-3p-enhanced pathogenicity coincided with the MIR337-3p depression of VitD-dependent antimicrobial response of cytochrome P450, family 27, subfamily b, polypeptide 1 (CYP27B1)/Beta-defensin 4 (DEFB4A)/ cathelicidin antimicrobial peptide CAMP pathways. Surprisingly, single MIR337-3p species could specifically target both the *Toll-like receptor 4* (*TLR4*) and *signal transducer and activator of transcription 3* (*STAT3*) 3′-untranslated regions (UTRs) to depress the TLR4/MYD88 and STAT3 signals and impair either of the two signals inhibiting the VitD-dependent antimicrobial pathways in macrophages. Concurrently, human peripheral blood mononuclear cells (PBMCs) expressing high-level MIR337-3p exhibited a reduced ability of innate cell populations to mount fast-acting cellular immunity against intracellular mycobacterial infection. Furthermore, a higher expression of Mir337-3p after mycobacterial infection of mice coincided with much greater colony-forming unit (CFU) counts in lungs and even the death of infected animals, whereas Mir337-3p inhibitor treatment of infected mice reduced Mir337-3p levels and reversed Mir337-3p-mediated increases in CFU counts. Thus, TB-driven single MIR337-3p species could specifically target/impair both TLR4/MYD88 and STAT3 activation signals, inhibiting VitD-dependent antimicrobial response and fast-acting anti-TB immunity, leading to enhanced pathogenicity.

## Introduction

Tuberculosis (TB), caused by *Mycobacterium tuberculosis* (*Mtb*), is the top 1 cause of death among infectious diseases worldwide. In 2018, there were about 10 million new TB cases, and 1.2 million deaths (1). *Mtb* is spread or transmitted *via* aerosol from active TB patients, and the bacilli can survive or grow in lung macrophages or epithelial cells of susceptible hosts (2). The balance between host immune response and TB infection determines the potential clinical outcomes of the resulting active TB, latent TB infection (LTBI), or resister status with early clearance of TB bacillus without immune signature of infection (3). Accordingly, the fates of TB bacilli in host macrophages are growth, dormancy, or elimination in respective hosts. Vitamin D (VitD) receptor (VDR)-related signaling pathways enhance antimicrobial defense *via* the generation of the antimicrobial peptides and play a key role in elimination of mycobacteria or *Mtb* in macrophages (4). Concurrently, emerging evidence suggests that innate-like T-cell populations including antigen (Ag)-specific Vγ2Vδ2 cells or natural killer (NK)/innate lymphoid cells (ILCs) may mount fast-acting antimycobacterial immunity for early clearance of bacilli (5–7).

MicroRNAs (miRNAs) regulate posttranscriptional silencing of target genes, and large amounts of miRNAs were upregulated or downregulated during *Mtb* infection as identified by miRNA sequencing (8). Upregulation of miRNAs influences processes such as autophagy and apoptosis during mycobacterial infection (9, 10). It has also been shown that MIRlet-7 targets tumor necrosis factor (TNF)-α-induced protein 3, a feedback inhibitor of the nuclear factor (NF)-κB pathway, and its downregulation helps depress innate immune responses against *Mtb* in macrophages (11). MIR27a targets endoplasmic reticulum (ER)-located Ca^2+^ transporter (calcium voltage-gated channel auxiliary subunit α2δ3, CACNA2D3) to inhibit autophagosome formation for promoting the intracellular survival of *Mtb* (12). While MIR337-3p plays a role in the regulation of proliferation, apoptosis, and cell viability (13), our previous work showed that MIR337-3p was highly expressed in γδ T cells of TB patients (14). However, it remains unknown whether MIR337-3p plays roles in the regulation of VDR-mediated antimicrobial response or fast-acting anti-TB immunity against *Mtb*, leading to an increased mycobacterial infection and TB disease.

It is unknown whether mycobacterium-driven single miRNA species can enhance TB pathogenicity by specific impairing of multiple signals for broad destruction of VDR-mediated antimicrobial response and fast-acting anti-TB immunity. This hypothesis was tested in the current study. Our results suggest that single MIR337-3p enhances mycobacterial pathogenicity by specific targeting/depressing both Toll-like receptor 4 (TLR4)/myeloid differentiation primary response protein MYD88 (MYD88) and signal transducer and activator of transcription 3 (STAT3) signals, reducing VDR-mediated antimicrobial response and fast-acting immunity.

## Materials and Methods

### Human Subjects

The study was approved by both the institutional review boards for human subject research and the institutional biosafety committees at Shanghai Pulmonary Hospital (SPH) of Tongji University. All subjects are adults and signed written informed consents.

TB patients were recruited at SPH (Shanghai, China). Age- and sex-matched uninfected volunteers without clinical and immunological evidence of TB or LTBI were recruited as healthy control (HC). All participants were tested for human immunodeficiency virus (HIV), hepatitis C virus (HCV), and hepatitis B virus (HBV). Individuals with HIV, HCV, and HBV infection and other infectious diseases or cancers were excluded.

In these experiments, we did not use any statistical methods to predetermine sample size, since the experiments were not randomized and the investigators were not blinded to allocation during experiments and outcome assessment (15).

### Peripheral Blood Mononuclear Cell Isolation and Quantitative Polymerase Chain Reaction Analysis for Gene Expression

Peripheral blood mononuclear cells (PBMCs) were isolated from ethylene diamine tetraacetic acid (EDTA)-treated blood using Ficoll-Paque Plus density gradient centrifugation and cultured in RPMI 1640 media supplemented with 2 mM glutamine, 50 U/ml penicillin, and 50 μg/ml streptomycin and containing 10% fetal bovine serum (FBS; Invitrogen) (14, 16).

Total ribonucleic acid (RNA) was extracted from PBMCs, freshly isolated cell subsets, or mouse lung using RNA column enrichment procedures (Cat: R1051, ZymoResearch, CA). CD14^+^, CD8^+^, CD4^+^, and Vδ2^+^ T cells were isolated and enriched from fresh PBMC using magnetic activated cell sorting (MACS) methods. An equivalent of 1 μg total RNA was removed from genomic DNA, followed by reverse transcription into cDNA using PrimeScript RT Reagent (Cat: RR047A, TaKaRa, Japan). The cDNA was used to amplify target gene fragment in triplicate reactions for each gene. Subsequently, quantitative polymerase chain reaction (qPCR) was performed using TB Green™ Premix Ex Taq™ II Reagent (Cat: RR820A, TaKaRa, Japan) on a QuantStudio™ 6 Flex Real-Time PCR System (Applied Biosystems, Foster City, CA, USA). The cycling conditions consisted of 95°C for 30 s, followed by 45 cycles of 95°C for 5 s, and 60°C for 30 s. Sequences of qPCR primers were designed using Primer3 or obtained from published literatures (**Table 1**). The housekeeping gene β-actin was used as internal control gene for normalization.

**Table 1:** Primers used for qPCR.

Gene	Forward primer	Reverse primer	Sources
*STAT3*	5′-TTTGAGACCGAGGTGTATCACC-3′	5′-GGTCAGCATGTTGTACCACAGG-3′	^§^Genbank Accession: NM_139276
*TLR4*	5′-CAGAGTTGCTTTCAATGGCATC-3′	5′-AGACTGTAATCAAGAACCTGGAGG-3′	(9)
*MYD88*	5′-GAGCGTTTCGATGCCTTCAT-3′	5′-GTTTGTCTGTTCCAGTTGCCG-3′	^§^Genbank Accession: NM_001172569
*VDR*	5′-CTGACCCTGGAGACTTTGAC-3′	5′-TTCCTCTGCACTTCCTCATC-3′	(17)
*CAMP*	5′-AGGATTGTGACTTCAAGAAGGACG-3′	5′-GTTTATTTCTCAGAGCCCAGAAGC-3′	(10)
*CYP27B1*	5′-GGAACCCTGAACAACGTAGTC-3′	5′-AGTCCGAACTTGTAAAATTCCCC-3′	^§^Genbank Accession: NM_000785
*DEFB4A*	5′-GGT GTT TTT GGT GGT ATA GGC G-3′	5′-AGG GCA AAA GAC TGG ATG ACA-3′	(18)
^‡^m*Stat3*	5′-GAAAAGGACATCAGTGGCAAGA-3′	5′-CGGGGTAGAGGTAGACAAGTGG-3′	^§^Genbank Accession: NM_011486
^‡^mβ*-Actin*	5′-GCCCTGAGGCACTCTTCCA-3′	5′-TGTTGGCGTACAGGTCTTTGC-3′	^§^Genbank Accession: NM_007393
β-*ACTIN*	5’-GCCCTGAGGCACTCTTCCA-3’	5’-TGTTGGCGTACAGGTCTTTGC-3’	^§^Genbank Accession: NM_001101

^‡^“m” means primers of mouse genes.

^§^Primers were designed using Primer3 and Blast in NCBI based on these sequences.

STAT3, signal transducer and activator of transcription 3; TLR4, toll like receptor 4; MYD88, myeloid differentiation primary response protein; VDR, Vitamin D receptor; CAMP, cathelicidin antimicrobial peptide; CYP27B1, cytochrome P450, family 27, subfamily b, polypeptide 1; DEFB4A, Beta-defensin 4.

The data were analyzed using the 2^-ΔΔCt^ method to calculate the relative expression of the target gene. First, the difference between the Ct values (ΔCt) of the gene of interest and the housekeeping gene is calculated for each experimental sample. Then, the difference in the ΔCt values between the experimental and control samples ΔΔCt is calculated. The fold change in the expression of the gene of interest between the two samples is then equal to 2^-ΔΔCt^. The relative expression levels of target mRNAs were normalized by β-actin, and the data from control groups were used as calibrator (19).

Total miRNA was extracted from freshly isolated PBMC or cell subsets using the miRcute miRNA isolation kit (Cat: DP501, Tiangen, Beijing, China). Relevant cDNA was synthesized with miRcute miRNA First-Strand cDNA Synthesis Kit (Cat: kr211-02, Tiangen, Beijing, China). qPCR primers of miR-337-3p (Cat : CD201-0356, Tiangen, Beijing, China) and U6 (Cat: CD201-0145, Tiangen, Beijing, China) were purchased from company. qPCR was performed using the miRcute Plus miRNA qPCR Kit (SYBR Green) (Cat: FP411-02, Tiangen, Beijing, China). The relative expression level of miRNA was normalized by U6.

### Mycobacteria Strains and Cell Culture

The *M. tuberculosis* H37Rv, *M. bovis* Bacillus Calmette-Guerin (BCG) Danish strain (ATCC35733), and *M. smegmatis* [ATCC700084/mc (2)155] were grown at 37°C in Difco Middlebrook 7H9 broth (Cat: 90003-876, Becton Dickinson) or on Middlebrook 7H10 agar (Cat: 90003-728, Becton Dickinson) supplemented with 10% oleic acid-albumin-dextrose-catalase-enriched Middlebrook (OADC, Cat: 90000-418, Becton Dickinson), 0.2% glycerol, and 0.05% Tween-80. Slow-growing H37Rv and BCG reach logarithmic phase for 3–4-week culture. *M. smegmatis* strains grow fast and reach logarithmic phase for 2–3-day culture. Cells or cultures were measured for mycobacterial colony counts in the biosafety level (BSL)-II+ facility (BSL3-equivalent lab for handling aerosol-free samples from patients) in SPH of Tongji University.

The human alveolar epithelial cell line A549 (RRID : CVCL_0023), human macrophage THP-1 (RRID: CVCL_0006), and mouse macrophage RAW264.7 (RRID : CVCL_0493) were grown in RPMI 1640 medium. VitD3 active form 1,25-(OH)2-vitamin D3 (CAS: 32222-06-3, Sigma-Aldrich) was used to stimulate cells at working concentration of 100 nM for 3 days or any given time.

THP-1 cells with concentration of 1 million per ml were treated with 50 ng/ml phorbol 12-myristate 13-actate (PMA, CAS: 16561-29-8, Sigma-Aldrich) for 48 h to differentiate into macrophages, then washed twice with phosphate buffered saline (PBS) and maintained for further infection or transfection.

Lipopolysaccharide (LPS, Cat: SMB00610, Sigma-Aldrich) was used to stimulate PMA-treated THP-1 cells at working concentration of 100 ng/ml for 6 h or any given time, and then cells were collected and used to isolate mRNA for qPCR.

Human monocytes of CD14^+^ cells were isolated and enriched from fresh PBMCs of healthy uninfected donors using MACS methods. Human monocyte-derived macrophages (hMDMs) were differentiated from fresh isolated CD14^+^ cells in RPMI 1640 medium, supplemented with L-glutamine (2 mM), sodium pyruvate (1 mM), 10% heat-inactivated FBS, and 50 ng/ml human M-CSF (Novoprotein) for 7 days.

### Cell Transfection

The microRNAs from human of MIR337-3p mimics (sequence: CUCCUAUAUGAUGCCUUU CUUC), MIR337-3p inhibitors (sequence: GAAGAAAGGCAUCAUAUAGGAG), and their counterparts in mice of Mir337-3p mimics (sequence: UCAGCUCCUAUAUGAUGCCUU U), Mir337-3p inhibitors (sequence: AAAGGCAUCAUAUAGGAGCUGA), and their control sequences were synthesized by a commercial company (Genechem, China) with store concentration of 20 μM, and the working concentration was 50 nM.

To change the expression level of MIR337-3p/Mir337-3p, A549, RAW264.7, and PMA-treated THP-1 cells were transfected with MIR337-3p/Mir337-3p mimics or inhibitors and controls, respectively, with Lipofectamine Transfection Reagent (Cat: L3000001, Thermo Fisher Scientific, USA) according to each manufacturer’s protocol. Cells were seeded in six-well plate with 1 × 10^6^ cells per well and were added with DNA–lipid complex of diluted DNA (synthesized microRNA mimics, inhibitors, and controls) with Lipofectamine Reagent when cells were 70%–90% confluent. The transfected cells were incubated for 2 days at 37°C. Then, cells were used to analyze target gene expression or infected by mycobacteria.

### Mouse Infection and Administration of Mir337-3p Inhibitors

C57BL/6 mice were purchased from the Shanghai Laboratory Animal Center. All mice were housed in specific pathogen-free conditions. All experiments were carried out according to the guide for the care and use of laboratory animals with the approval of the institutional animal care and use committee of SPH of Tongji University.

Mice were challenged by tail vein injection with 4 × 10^7^ colony-forming unit (CFU) BCG in 200 µl PBS buffer per mouse (4–6 weeks of age) at 0 and 20 days. On the fourth day, mice were administered lentivirus expressing Mir337-3p inhibitor (Genechem, China) at 5 × 10^6^ TU per mouse in hind footpad (20). Empty lentivirus was injected into control mice. Twenty-eight days after the first BCG injection, mice were sacrificed, and samples were harvested. The lungs were homogenized in PBS and plated on 7H10 agar to count BCG CFUs.

### Dual-Luciferase Reporter Assay

Wild-type or mutant fragments of 3′-untranslated region (UTR) of *TLR4* were constructed and inserted downstream of the luciferase reporter gene of the PGL3-CMV-LUC-MCS (RRID: Addgene_17186, Genomeditech, Guangzhou, China). Transgene reagent was used to transfect the reporter plasmids into HEK-293 cells (RRID : CVCL_0045). Renilla luciferase assay was used to detect luciferase activity according to technical manual of Dual-Luciferase Reporter Assay System (Promega, China). Relative light unit (RLU) was tested. The RLU values of mimics control group were taken as 1, and the relative values in other groups were calculated.

### Mycobacteria Infection of Host Cells

Cells of A549, PMA-treated THP-1, and RAW264.7 were infected with BCG at a multiplicity of infection (MOI) of 10 for ~4 h. Cells of A549, RAW264.7, and hMDM were infected with H37Rv at a MOI = 2 for 4 h. In some experiments, cells were firstly transfected with microRNA mimics or inhibitors and controls as described above and then infected with BCG, H37Rv, or *M. smegmatis* strains.

After infection, extracellular bacilli were removed by washing four times with PBS buffer, and the infected macrophages and lung epithelial cells were cultured for 3 days or given time. Then, the infected cells were lysed in sterile PBS with sodium dodecyl sulfate (SDS; Sigma-Aldrich) in a working concentration of 0.3 g/L. Serial dilutions were performed for quantitative culturing. Mycobacteria viability was quantified *via* counting CFUs as we previously described (19).

### Western Blotting

Cells were transfected with miR-337-3p mimics or inhibitors and controls and stimulated by VitD3 (100 nM), LPS (100 ng/ml), and medium overnight, respectively. Then, cells were lysed by incubation in radioimmunoprecipitation assay (RIPA) lysis buffer on ice for 5 min. Next, lysates were separated by SDS polyacrylamide gel electrophoresis (SDS-PAGE) and transferred to a polyvinylidene difluoride membrane (Merck/Millipore). After blocking with 50 g/L bovine serum albumin (BSA), the membrane was incubated with antibodies (Abs) against STAT3 (RRID : AB_331757, Cat: 9139S, Cell Signaling Technology), TLR4 (RRID : AB_300457, Cat: ab13556, Abcam), MYD88 (Cat: RRID : AB_10547882, Cat: 4283S, Cell Signaling Technology), CYP21B1 (RRID : AB_10672635, Cat: ab95047, Abcam), or ACTIN (RRID : AB_2687938, Cat: 66009-1, Proteintech) overnight at 4°C, followed by incubation with the respective secondary antibodies.

Image Lab software (Bio-Rad Laboratories, USA) was used to capture and analyze digital image data from electrophoresis gels and blots. The ratio of target proteins and actin in control groups was taken as 1, and the values of the ratios of target proteins and actin in experimental groups compared to the ratios in control groups were used to represent the target protein relative expression levels.

### Statistical Analysis

Statistical analysis was done by using GraphPad Prism software (GraphPad Software, CA). P < 0.05 was considered to indicate a statistically significant difference. The difference of miRNA/gene expression between treatment and control groups was evaluated using paired *t*-test. One-way analysis of variance (ANOVA) followed by Bonferroni *post-hoc* test was used for multiple comparisons. Mycobacteria CFU counts were analyzed using the Mann–Whitney U test (21).

## Results

### MicroRNA MIR337-3p/Mir337-3p Was Highly Expressed in Tuberculosis Patients and *Mycobacterium*-Infected Mice

**Figure 1:**
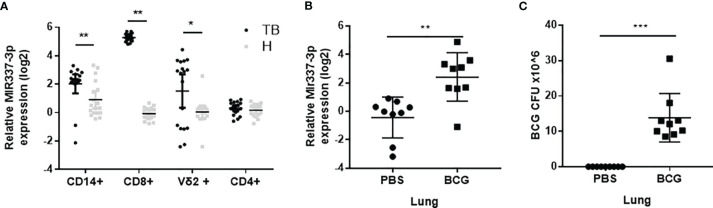
MicroRNA MIR337-3p/Mir337-3p was highly expressed in lymphocytes of tuberculosis (TB) patients and in lungs of mycobacterium-infected mice. **(A)** The relative expression levels of MIR337-3p in CD14^+^, CD8^+^, and Vγ2^+^ T cells in TB patients (n = 18) were significantly higher than the counterparts in healthy controls (HCs, n = 18), as measured by quantitative polymerase chain reaction (qPCR). **(B)** The expression of Mir337-3p in lungs of *M. bovis* BCG-infected mice were higher than those in phosphate buffered saline (PBS)-infected animals (n = 9). **(C)** The *M. bovis* Bacillus Calmette-Guerin (BCG) infection levels in lungs of BCG-infected mice (n = 9). Data are represented as mean ± standard errors. *P < 0.05, **P < 0.01, and ***P < 0.001.

Our previous studies suggest that TB patients exhibited high expression levels of MIR337-3p in phosphoantigen-specific γδ T cells (14). However, the role of mycobacterium-driven MIR337-3p in TB infection remains unclear. As an initial effort to address this question, we comparatively examined expression levels of MIR337-3p in CD14^+^ monocytes/macrophages, CD8^+^ lymphocytes containing NK/innate lymphoid cells, innate-like Vγ2^+^ T cells, and adaptive CD4^+^ T cells between TB patients and HC subjects. We found that MIR337-3p was highly upregulated in CD14^+^ monocytes, CD8^+^ lymphocytes, and Vγ2^+^ T cells, but not CD4^+^ T cells, in TB patients, with about 5-, 40-, and 10-fold increases, respectively, when compared to the counterparts of HCs (**Figure 1A**). Since these cell populations play balancing or protective roles in the antimicrobial response, cytotoxic killing, and innate/adaptive immunity (5, 9, 15), the increased MIR337-3p in these cells might have negative impacts on immune responses against TB infection.

We then sought to determine whether mycobacterial infection could upregulate the Mir337-3p expression. We did a proof-of-concept experiment in which mice were infected with high-dose *M. bovis* BCG or PBS control and then euthanized and assessed for Mir337-3p expression levels in the lungs at 3 weeks after infection. The use of BCG here was justified for proving a concept in the acute experiment, rather than for inducing chronic TB infection or pathology. We found that the expression levels of Mir337-3p in lungs of BCG-infected mice were >5 times higher than those of PBS control mice (**Figure 1B**). Moreover, such high levels of Mir337-3p in lungs were coincident with BCG infection burdens (**Figures 1B, C** **)**.

Together, the results above demonstrated that MIR337-3p/Mir337-3p was highly expressed in TB patients and in mycobacterium-infected mice, and that increases in Mir337-3p correlated with mycobacterial infection burdens.

### High-Level MIR337-3p Expression Was Induced by Mycobacterial Infection in Cellular Models

**Figure 2:**
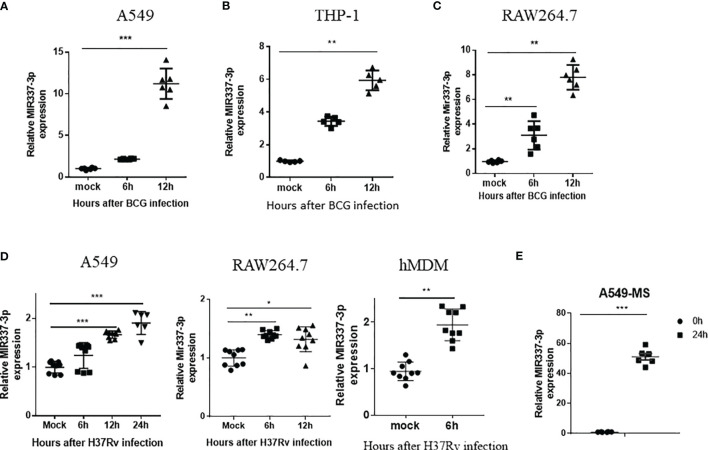
High-level MIR337-3p/Mir337-3p expression was induced by mycobacterial infection in cellular models. **(A–C)** show that BCG infection induced a significantly higher expression of MIR337-3p/Mir337-3p in lung alveolar epithelial A549 cells **(A)** and macrophages THP-1 **(B)** and RAW264.7 **(C)**. Cells were infected by *M. bovis* BCG (MOI = 10) for 0, 6, and 12 h, respectively. The expression of MIR337-3p/Mir337-3p was measured by qPCR. **(D)** shows that the virulent strain of *M. tuberculosis* H37Rv infection (MOI = 2) enhanced MIR337-3p expression in human epithelial A549 cells, mouse macrophage RAW264.7, and human monocyte-derived macrophages (hMDMs). **(E)** shows that fast-growing *M. smegmatis* (MS) infection also induced high-level MIR337-3p expression in A549 cells. Data are shown as mean ± standard errors. *P < 0.05, **P < 0.01, and ***P < 0.001.

Because TB patients exhibited an increased MIR337-3p in *Mtb* bacillus target cells (CD14^+^ monocytes/macrophages), we sought to determine whether mycobacterial infection could directly induce MIR337-3p expression. To this end, macrophages and lung epithelial cells were infected with BCG or *M. tuberculosis* H37Rv (14, 19, 22) and assessed for MIR337-3p expression. BCG infection of lung epithelial A549 cells led to >10 times greater expression of MIR337-3p at 12 h after infection compared to medium control (**Figure 2A**). Similarly, BCG infection of macrophage THP-1 and RAW264.7 cells resulted in six and eight times greater expressions of MIR337-3p/Mir337-3p, respectively, at 12 h after infection (**Figures 2B, C** **)**. Consistently, the virulent strain of *M. tuberculosis* H37Rv infection also significantly enhanced MIR337-3p/Mir337-3p expression in human epithelial A549 cells (**Figure 2D**, left panel), mouse macrophages of RAW264.7 (**Figure 2D**, middle panel), and hMDMs (**Figure 2D**, right panel) despite that the dose for *Mtb* exposure/infection was really low (MOI = 2). Furthermore, fast-growing *M. smegmatis* infection also induced high-level MIR337-3p expression in A549 cells (**Figure 2F**). These results support the notion that mycobacterial infection of lung epithelial cells, macrophage cell lines, and hMDM cells can directly induce the MIR337-3p expression.

### MIR337-3p Promoted the Mycobacterial Entry/Infection and Replication/Growth in Host Target Cells

**Figure 3:**
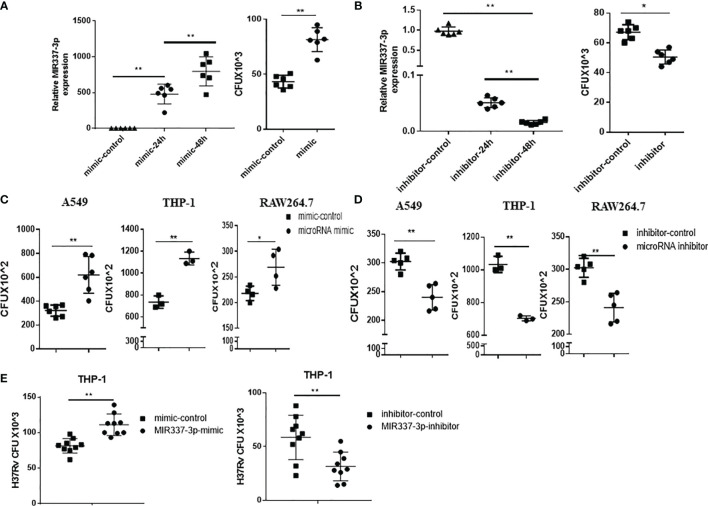
MIR337-3p/Mir337-3p promoted the mycobacterial entry/infection and replication/growth in host target cells. The target cells were transfected with MIR337-3p mimics or inhibitors and their controls, respectively, and cocultured with BCG for 4 h or 3 days and then lysed and grown in plates to count BCG CFUs. **(A)** shows that MIR337-3p expression was markedly increased in THP-1 cells transfected with MIR337-3p mimics compared to the controls at 24 and 48 h after transfection (left panel), and the BCG CFU counts in the 24-h mimic-transfected THP-1 cells were significantly higher than those in the mimic control-transfected cells after 4-h BCG coculture (right panel). **(B)** shows that inhibitor transfection decreased MIR337-3p expression at 24 and 48 h after transfection (left panel) and also decreased the BCG CFU counts in 24-h inhibitor-transfected cells after 4-h BCG coculture (right panel). **(C, D)** show BCG CFU counts in A549, THP-1, and RAW264.7 cells 24 h-transfected with MIR337-3p/Mir337-3p mimics/controls and inhibitors/controls, respectively, at 3 days of culture after 4-h-BCG incubation. **(E)** shows virulent H37Rv CFU counts in THP-1 cells transfected with MIR337-3p mimics/controls or inhibitors/controls, respectively, at 3 days of culture after H37Rv infection. *P < 0.05 and **P < 0.01.

Given the strong correlation between mycobacterial infection and MIR337-3p expression (**Figures 1**, **2**), we sought to address the concept that MIR337-3p can promote mycobacterial infection. To this end, we utilized MIR337-3p mimics and MIR337-3p inhibitors to increase and knock down MIR337-3p, respectively, and then determined whether such changes altered mycobacterial entry/infection and replication/growth. At 24 h after transfection of MIR337-3p mimics into THP-1 cells, MIR337-3p increased by >500 times compared to the control (**Figure 3A**, left panel). These transfected THP-1 cells with high-level MIR337-3p were cocultured for 4 h with BCG at MOI = 10, then washed intensely to remove free unbound BCG, and then lysed for measurements of BCG CFU counts. BCG CFU counts in the mimics-transfected THP-1 cells were significantly higher than those in the control (**Figure 3A**, right panel). Results in such 4-h short-time experiments suggested that MIR337-3p promoted BCG entry/infection. Concurrently, while the transfection of MIR337-3p inhibitors knocked down >95% miR-337-3p at 48 h (**Figure 3B**, left panel), the inhibitor-transfected THP-1 cells exhibited significantly lower BCG CFU counts than the controls (**Figure 3B**, right panel). These results implicated that the knockdown of MIR337-3p by the inhibitors reversed MIR337-3p-induced increases in BCG entry/infection in the target cells after 4-h coculturing with BCG.

We then determined whether MIR337-3p/Mir337-3p could enhance BCG replication/growth at 3 days after 4-h BCG coculturing of MIR337-3p/Mir337-3p-manipulated A549, THP-1, or RWA264.7 cells, which were transfected 24 h earlier with MIR337-3p/Mir337-3p mimics or treated 48 h earlier with inhibitors. At 3 days after BCG coculturing for 4 h, transfected cells were lysed to measure BCG CFU counts. We found that BCG CFU counts in MIR337-3p/Mir337-3p mimics-transfected cells were significantly higher than those in the controls (**Figure 3C**). Consistently, BCG CFU counts in the inhibitor-treated cells were significantly lower than those in the controls (**Figure 3D**). Moreover, the virulent strain of *M. tuberculosis* H37Rv CFU counts in MIR337-3p mimics-transfected THP-1 cells were also higher than those in the controls (**Figure 3E**, left panel), and H37Rv CFU counts in the inhibitor-treated cells were lower than those in the controls (**Figure 3E**, right panel). These results therefore demonstrated that MIR337-3p/Mir337-3p could enhance BCG or *Mtb* H37Rv replication/growth in lung epithelial A549 cells and THP-1/RWA264.7 macrophages.

Together, the data above suggest that while mycobacterial infection induces high-level MIR337-3p/Mir337-3p, MIR337-3p/Mir337-3p can promote the mycobacterial entry/infection and replication/growth in host target cells.

### MIR337-3p Depressed Antimicrobial Response of Vitamin D Receptor-Related CYP27B1, DEFB4A, and CAMP Pathways

Then, we conducted additional in-depth mechanistic experiments to examine mechanisms whereby MIR337-3p can depress innate immunity against mycobacterial infection. Our results above already established the following points: (i) mycobacterial infection led to high-level MIR337-3p; (ii) using MIR337-3p mimics and inhibitors to manipulate MIR337-3p increase and knockdown, respectively, we confirmed that MIR337-3p promoted mycobacterial infection.

Based on these results, we determined if MIR337-3p perturbed innate antimycobacterial responses in target cells for enhanced pathogenicity of mycobacterial infection. Data from our cellular models of initial infection implicate that the MIR337-3p augments mycobacterial infection by disrupting antimicrobial response or fast-acting innate-like immunity against mycobacterial infection (23, 24). Since VDR-related antimicrobial activities involve CYP27B1 (cytochrome P450, family 27, subfamily b, polypeptide 1), DEFB4A (Beta-defensin 4A), and CAMP (cathelicidin antimicrobial peptide, also named LL-37) pathways in macrophages, we tested the hypothesis that MIR337-3p could broadly inhibit CYP27B1, DEFB4A, and CAMP pathways, leading to an enhanced mycobacterial infection.

**Figure 4:**
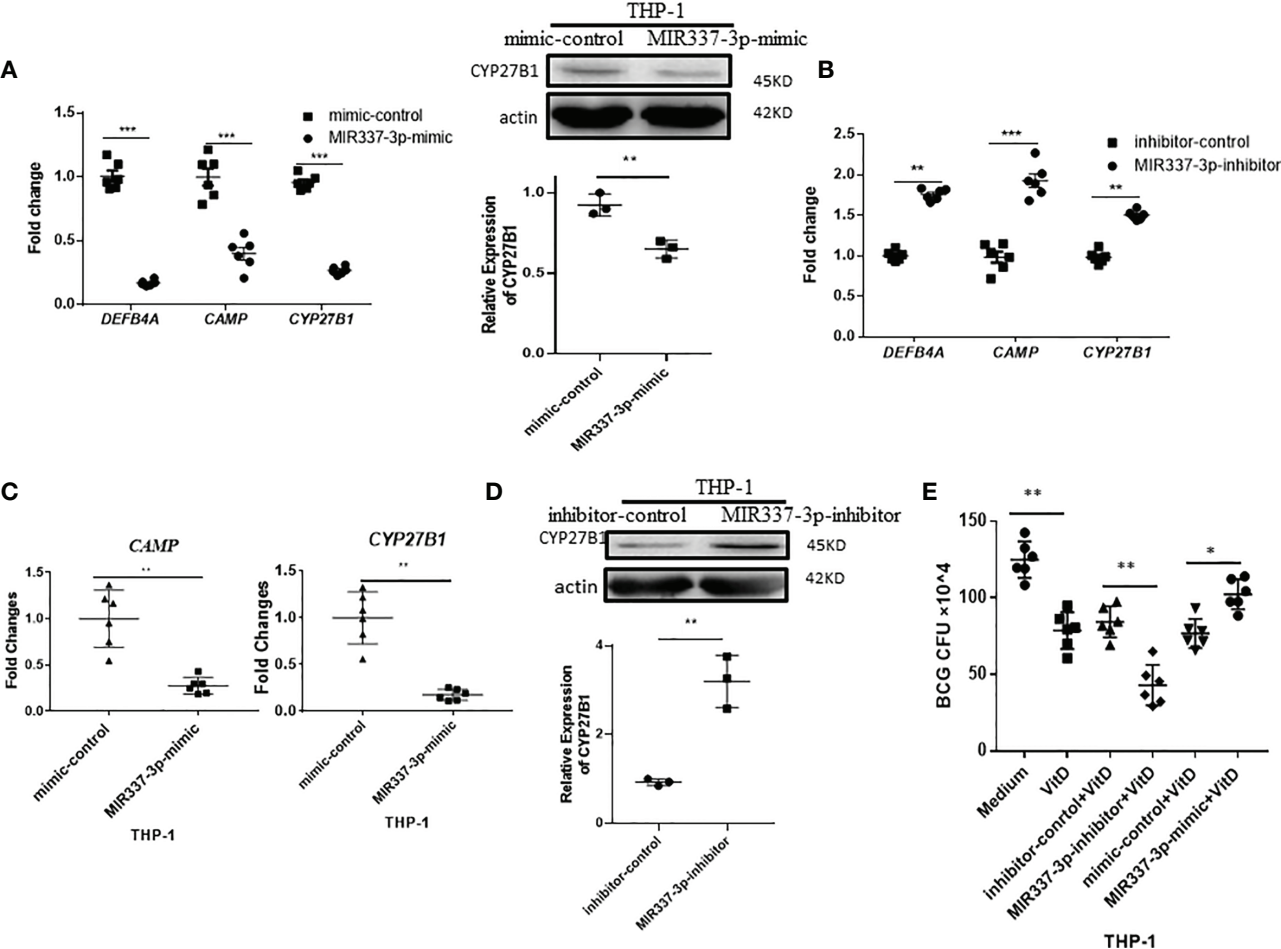
MIR337-3p depressed antimicrobial response of VDR-related CYP27B1, DEFB4A, and CAMP pathways. **(A)** Transfection of MIR337-3p mimics downregulated gene expression of *DEFB4A*, *CAMP*, and *CYP27B1* in THP-1 cells at 24 h after transfection, respectively (left panel). And transfection of MIR337-3p mimics depressed CYP27B1 protein expression in THP-1 cells (right panel). **(B)** The gene expressions of *DEFB4A*, *CAMP*, and *CYP27B1* were higher in MIR337-3p inhibitor-transfected THP-1 cells than those in inhibitor-control-transfected cells, respectively. **(C)** shows relative gene expression levels of *CAMP* and *CYP27B1* in MIR337-3p mimic- and control-transfected cells in stimulation of vitamin 1,25-(OH)2D3, respectively. **(D)** shows representative results in Western blot (WB) indicating that MIR337-3p inhibitor enhances CYP27B1 protein expression in THP-1 cells in stimulation of vitamin 1,25-(OH)2D3. **(E)** shows BCG CFU counts in vitamin 1,25-(OH)2D3-treated THP-1 cells, which were transfected with MIR337-3p mimics or inhibitors and their controls, respectively. Data are represented as mean ± standard errors. *P<0.05, **P < 0.01 and ***P < 0.001.

We transfected macrophages with specific MIR337-3p mimics and inhibitors and then measured altered expression levels of *CYP27B1*, *DEFB4A*, and *CAMP*. We found that expressions of *CYP27B1*, *DEFB4A*, and *CAMP* in cells transfected with MIR337-3p mimics were significantly decreased compared to the controls (**Figure 4A**, left panel). Also, the CYP27B1 protein expression levels in MIR337-3p mimics-transfected THP-1 cells were significantly downregulated (**Figure 4A**, right panel). Consistently, when MIR337-3p inhibitors were transfected into macrophages, the expressions of *CYP27B1*, *DEFB4A*, and *CAMP* were significantly increased compared to the controls (**Figure 4B**). Since VitD stimulates and activates antimicrobial pathways, we sought to examine whether miR-337-3p could reduce the VitD-mediated antimicrobial signaling in the VitD-treated cells. To this end, we further treated MIR337-3p-transfected macrophages with VitD and measured depression of *CYP27B1* and *CAMP*. The expressions of *CYP27B1* and *CAMP* were reduced to about 15% and 20% of their counterparts in controls under VitD stimulation, respectively (**Figure 4C**). However, the Western blot results showed that CYP27B1 protein expressions in MIR337-3p inhibitor-transfected THP-1 cells were about three times more than those in inhibitor-control-transfected cells under VitD stimulation (**Figure 4D**). To examine whether MIR337-3p regulated VitD-related antimicrobial activity, we changed MIR337-3p expression levels in THP-1 cells through transfection with MIR337-3p mimics or inhibitors and the respective controls, then treated these cells with VitD after 4-h infection with BCG, and then cocultured them for 3 days before cell lysis was measured for BCG CFU. Interestingly, MIR337-3p mimic-treated group exhibited significantly higher BCG CFU counts than the control group (**Figure 4E**), suggesting that MIR337-3p overexpression suppressed VitD-mediated antimicrobial activity. Concurrently, the MIR337-3p inhibitor-treated group exhibited lower BCG CFU counts than those in the control group (**Figure 4E**), implicating that inhibitor downregulation of MIR337-3p reversed the MIR337-3p suppression of VitD antimicrobial activity. Together, the above results suggest that MIR337-3p could depress VitD-related antimycobacterial response of CYP27B1, DEFB4A, and CAMP pathways, leading to enhanced mycobacterial infection.

### MIR337-3p Targeted the *TLR4* 3′-UTR and Depressed the TLR4/MYD88 Signal, Reducing Vitamin D Receptor-Related Antimicrobial Response

**Figure 5:**
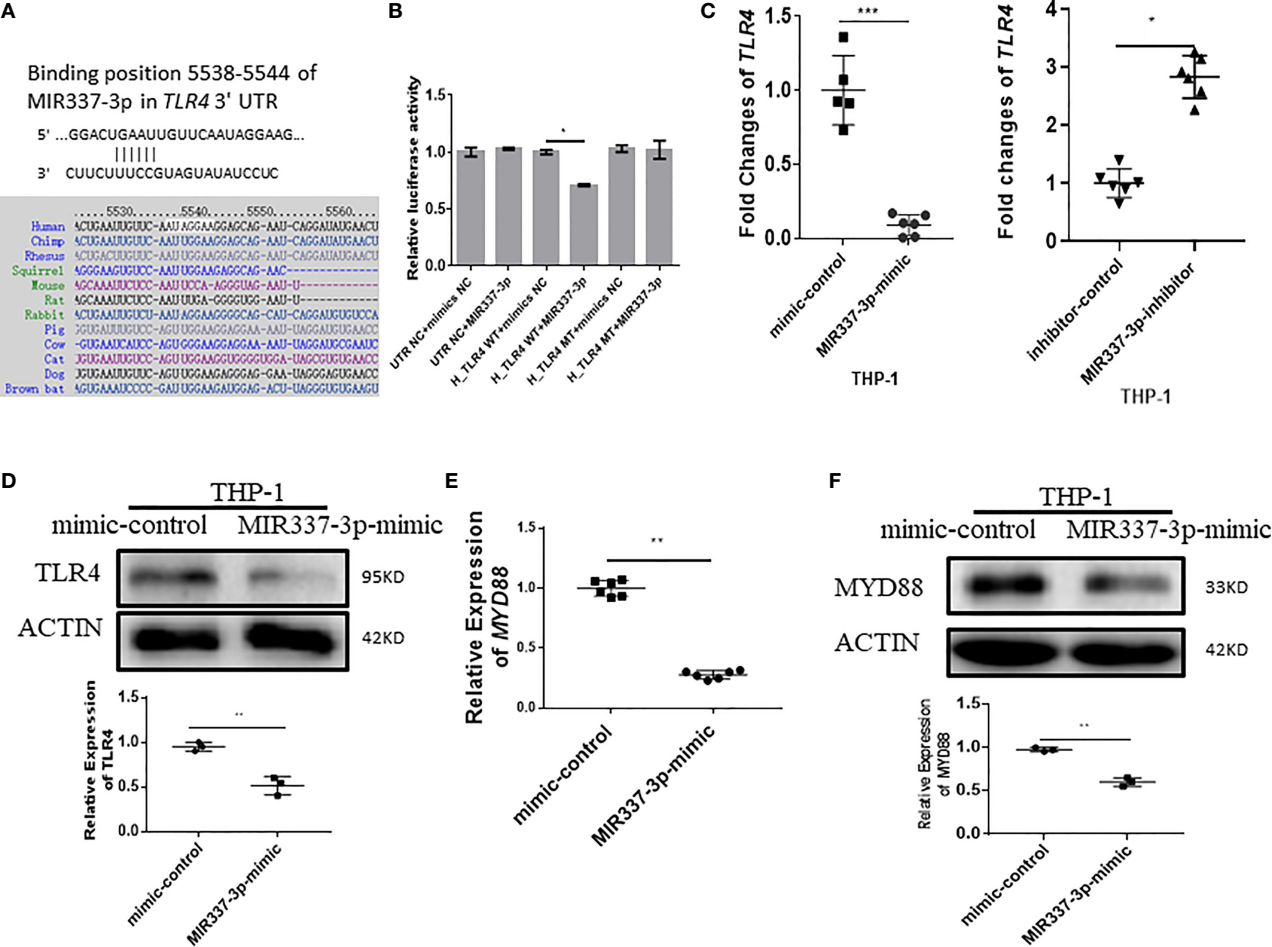
MIR337-3p targeted the *TLR4* 3′-UTR and depressed TLR4/MYD88 signal. **(A)** shows that the binding sites of MIR337-3p in *TLR4* 3′-UTR were predicted by TargetScan software. **(B)** shows data of luciferase reporter assays, which were performed to test the interaction of MIR337-3p and its target *TLR4* 3′-UTR using constructs containing the predicted targeting sequence (TLR4 WT) and mutant sequence (TLR4 MT) cloned into the 3′-UTR of the reporter gene. **(C)** shows that transfection of MIR337-3p mimics inhibits the expression of *TLR4* in THP-1 cells, and miR-337-3p inhibitors increased *TLR4* expression compared to the controls at 24 h after transfection. **(D)** shows the representative result of Western blot (WB) indicating that MIR337-3p mimics inhibit TLR4 protein expression in THP-1 cells. **(E)** shows that MIR337-3p mimics inhibit expressions of *MYD88* in THP-1 cells. **(F)** shows the representative WB result indicating that MIR337-3p mimics inhibit MYD88 protein expression in THP-1 cells. *P < 0.05, **P < 0.01, and ***P < 0.001.

We then sought to assess upstream pathways for the mechanism by which MIR337-3p regulates expressions of VitD-activated CYP27B1, DEFB4A, and CAMP. We screened and predicted the binding targets of MIR337-3p using TargetScan algorithm and found that there was potential binding site of MIR337-3p in *TLR4* 3′-UTR (**Figure 5A**). To confirm the interaction between MIR337-3p and *TLR4* 3′-UTR, we performed luciferase reporter assay. Results showed that relative luciferase activity was significantly decreased in the MIR337-3p-treated sample with wild-type (WT) *TLR4* 3′-UTR compared to controls (**Figure 5B**). And there was no difference between the relative luciferase activities of the MIR337-3p and control microRNA-treated samples with mutant-type (MT) *TLR4* 3′-UTR, in which the binding sites of MIR337-3p were mutated (**Figure 5B**). These results demonstrated that MIR337-3p could bind to *TLR4* 3′-UTR to inhibit target gene *TLR4* expression.

To confirm whether MIR337-3p affected TLR4 expression in cells, we transfected macrophages with specific MIR337-3p mimics, inhibitors, or controls and then examined altered expression levels of TLR4. The expression of *TLR4* in MIR337-3p mimic-transfected cells was significantly reduced compared to that of controls (**Figure 5C**). Concurrently, when MIR337-3p inhibitors were transfected into macrophages, the expressions of *TLR4* were significantly increased compared to those the controls (**Figure 5C**). Furthermore, we examined whether MIR337-3p altered TLR4 protein expression using MIR337-3p mimics. The Western blot results showed that TLR4 protein expression levels in MIR337-3p mimic-transfected THP-1 cells were about half of those in the controls (**Figure 5D** and **Supplementary Figure 1A**). Thus, these results implied that MIR337-3p could specifically regulate TLR4 expression in both transcription and translation levels.

Since MYD88 is a downstream essential adaptor protein in the TLR4 signaling pathway, we examined whether MIR337-3p-targeted reduction of TLR4 signaling also altered the expression of MYD88. We found that expression of *MYD88* in MIR337-3p-transfected THP-1 cells was only about one-third of that in controls (**Figure 5E**). Correspondingly, MYD88 protein expression was also significantly reduced in MIR337-3p-transfected THP-1 cells compared to that in controls (**Figure 5F** and **Supplementary Figure 1B**). The results demonstrate that MIR337-3p specifically targeted and regulated the TLR4–MYD88 signaling pathway.

**Figure 6:**
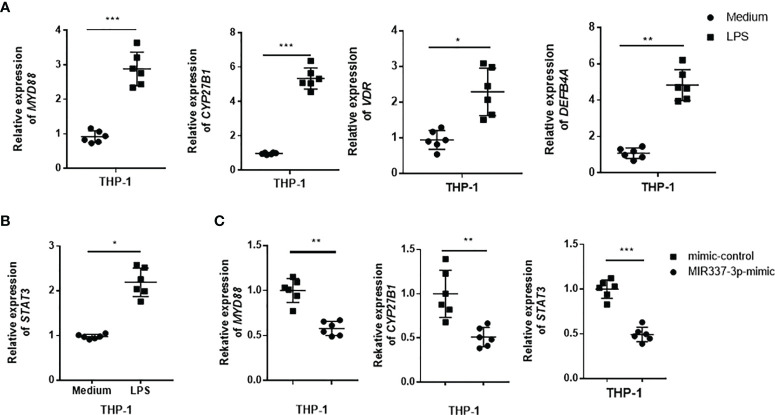
TLR4 signaling could activate VDR-related antimycobacterial pathways, and MIR337-3p could impair both the TLR4/MYD88 signal and the VDR-related antimicrobial response. **(A)** shows that LPS stimulation induced *MYD88*, *CYP27B1*, *VDR*, and *DEFB4A* expression in THP-1 cells. Medium was taken as control. **(B)** shows that LPS stimulation significantly upregulated *STAT3* expression in THP-1 cells. **(C)** shows that mimics of MIR337-3p inhibited the expression of *MYD88*, *CYP27B1*, and *STAT3* in THP-1 cells stimulated by LPS. Data are expressed as mean ± standard errors. *P < 0.05, **P < 0.01, and ***P < 0.001.

Next, we sought to examine whether TLR4/MYD88 signaling could activate VDR-related antimycobacterial pathways and whether MIR337-3p could impair the upstream TLR4/MYD88 signal and the downstream VDR antimicrobial response seen in **Figure 4**. To this end, THP-1 cells were stimulated with LPS, the TLR4 ligand capable of activating TLR4/MYD88 signaling pathway, and assessed for the expression of VDR antimicrobial signals. The results showed that LPS stimulation significantly increased the expression of MYD88 (**Figure 6A**), confirming the LPS activation of TLR4/MYD88 signaling pathway, as defined in literature (25). Surprisingly, the expressions of *VDR*, *CYP27B1*, and *DEFB4A* in LPS-stimulated cells were increased up to 5-fold compared to those of controls (**Figure 6A**), suggesting that LPS-stimulated TLR4/MYD88 signaling can activate the downstream VDR antimicrobial pathways. Of note, LPS stimulation could also activate another upstream signal STAT3 in macrophages (**Figure 6B**). Interestingly, despite LPS stimulating, MIR337-3p-transfected macrophages exhibited a significantly reduced expression of *MYD88* when compared to that of controls (**Figure 6C**). These results suggest that MIR337-3p overexpression can antagonize LPS activation of TLR4/MYD88 signaling pathway. Consistently, the miR-337-3p downregulation of MYD88 in the setting of LPS stimulation coincided with a decreased expression of CYP27B1, the key rate-limiting enzyme of VitD-activated antimicrobial pathways as well as the STAT3 signal, when compared to that of controls (**Figure 6C**).

Thus, the results above from in-depth mechanistic experiments demonstrated that MIR337-3p targeted/depressed upstream TLR4/MYD88 and STAT3 signaling and the downstream VDR antimicrobial pathways of CYP27B1/DEFB4A/CAMP, leading to an enhanced mycobacterial entry/infection and growth (**Figure 3**).

### MIR337-3p Depressed Either the TLR4/MYD88 or STAT3 Signal to Reduce the Vitamin D Receptor Antimicrobial Response in Macrophages

**Figure 7:**
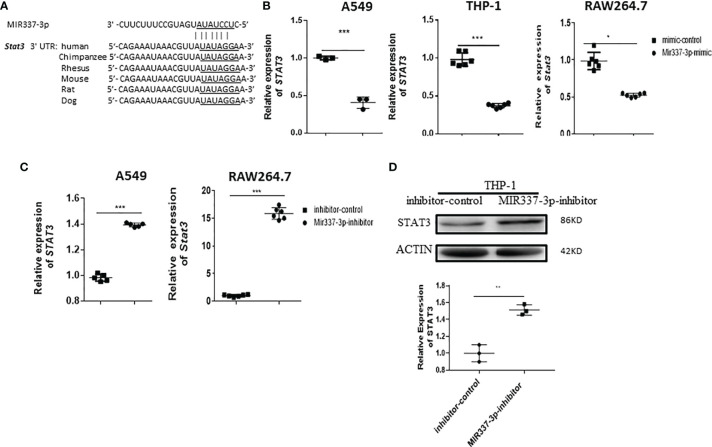
*Mycobacterium*-driven MIR337-3p could depress STAT3 signal by targeting *STAT3* 3′-UTR. **(A)** shows the binding sites of MIR337-3p in 3′-UTR of *STAT3* predicted by TargetScan software. **(B)** shows that transfection of MIR337-3p/Mir337-3p mimics led to downregulation of *STAT3/Stat3* in BCG-infected human lung alveolar epithelial A549 cells, human THP-1, and mouse RAW264.7 macrophages, respectively. **(C)** shows that transfection of MIR337-3p/Mir337-3p inhibitors increased the expression of *STAT3/Stat3* in BCG-infected A549 and RAW264.7 cells, respectively. **(D)** shows representative WB result indicating that MIR337-3p inhibitors enhanced STAT3 protein expression in THP-1 cells. Data are represented as mean ± standard errors. *P < 0.05, **P < 0.01, and ***P < 0.001.

Our previous work showed that a low level of STAT3 in phosphoantigen-specific γδ T cells was associated with MIR337-3p expression in TB patients (14). We also showed that MIR337-3p could also impair STAT3 signal in addition to TLR4/MYD88. These findings prompted us to perform computer model-based analysis of MIR337-3p binding to *STAT3* mRNA. The model analysis revealed that MIR337-3p could readily bind to the 3′-UTR of *STAT3* (**Figure 7A**), indicating that single MIR337-3p is able to target/bind to both the *STAT3* and *TLR4* 3′-UTRs (**Figures 5**, **7A**).

For proof of concept, we determined whether MIR337-3p could act as a potent regulator to downregulate *STAT3* expression. We measured changes in *STAT3* expression when the MIR337-3p expression was increased or decreased by manipulation in lung epithelial and macrophage cells. When MIR337-3p/Mir337-3p expression was increased by transfection of MIR337-3p/Mir337-3p mimics into A549, THP-1, and RAW264.7 cells, expression of *STAT3*/*Stat3* decreased by ~60%, ~65%, and ~50%, respectively, compared with that of control cells transfected with control nucleotides (**Figure 7B**). Concurrently, when MIR337-3p/Mir337-3p expression was knocked down by transfection of specific MIR337-3p/Mir337-3p inhibitors, the expression of *STAT3*/*Stat3* in inhibitor-transfected cells was ~15 times higher than that in controls (**Figure 7C**), and the STAT3 protein expression also highly increased in inhibitor-transfected cells compared to that in controls (**Figure 7D** and **Supplementary Figure 1C**). Importantly, our previous study established that lentivirus-shRNA silencing of STAT3 depressed antimicrobial activities of the VDR-related CYP27B1, DEFB4A, and CAMP pathways (7). Together, these results demonstrated that MIR337-3p, while targeting/depressing TLR4/MYD88, could also decrease STAT3 expression by binding the *STAT3* 3′-UTR and thus suppress the downstream VDR antimycobacterial pathways/activities.

Notably, LPS activation of TLR4/MYD88 signal significantly increased *STAT3* expression in LPS-stimulated THP-1 macrophages compared to that in controls (**Figure 6B**). It is also noteworthy that MIR337-3p overexpression could override the LPS/TLR4/MYD88 activation to reduce *STAT3* expression by ~50% in MIR337-3p-transfected cells when compared to that in controls (**Figure 6C**).

**Figure 8:**
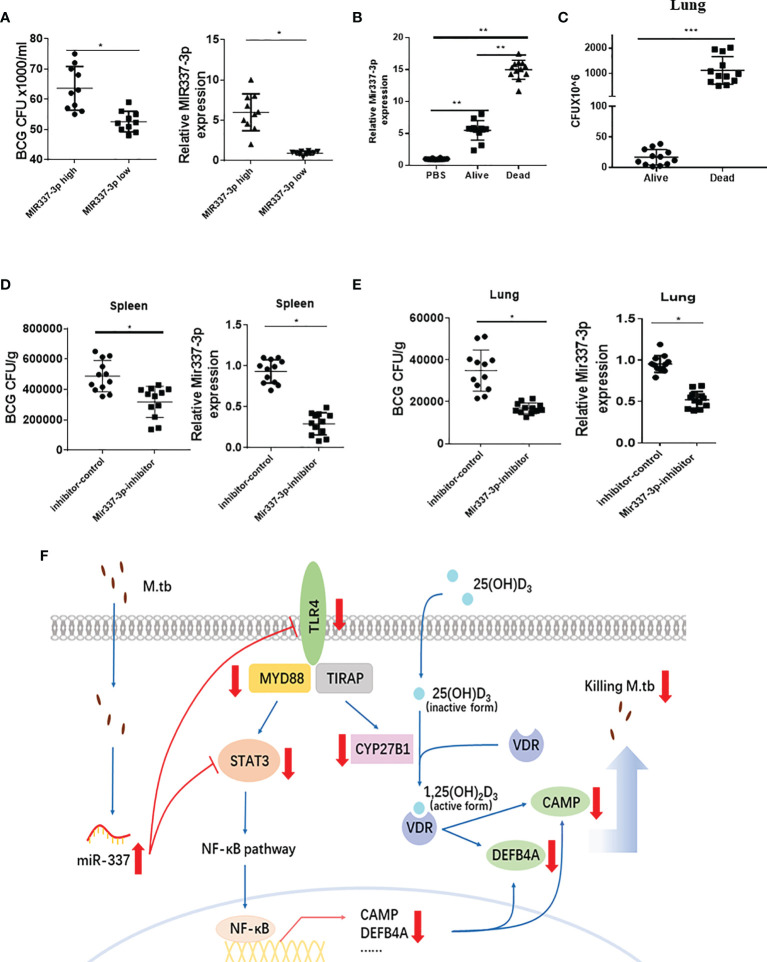
*Mycobacterium*-driven MIR337-3p/Mir337-3p reduced fast-acting cellular immunity against mycobacterial infection. **(A)** shows the BCG CFU counts in cocultures of BCG-infected A549 cells and PBMCs isolated from subjects expressing high and low MIR337-3p expression levels (left panel), respectively. Animals were divided into high and low MIR337-3p expression level groups (n = 10) (right panel). **(B)** shows relative Mir337-3p expression in lungs of PBS- and BCG-infected mice (n = 12). The BCG-infected mice were divided into alive and dead groups at 3 weeks after high dose of BCG (1 × 10^7^ CFU) challenge. **(C)** shows BCG CFU counts in lungs of alive and dead mice, respectively, at 3 weeks after BCG challenge. **(D)** shows BCG CFU counts (left panel) and Mir337-3p expression levels (right panel) in spleen of mice (n = 12) treated with Mir337-3p inhibitors (lentivirus construct) or controls at 3 weeks after BCG infection. **(E)** shows BCG CFU counts (left panel) and Mir337-3p expression levels (right panel) in lungs of mice (n = 12) treated with Mir337-3p inhibitors or controls. **(F)** A schematic model postulates that mycobacterial infection upregulates MIR337-3p expression and that MIR337-3p depresses *TLR4* and *STAT3* expression by binding their 3′-UTRs and inhibits the downstream CAMP, DEFB4A expressions, leading to impairing of VDR-mediated antimicrobial responses. *P < 0.05, **P < 0.01, and ***P < 0.001.

Together, the results above demonstrated that while LPS/TLR4/MYD88 activation augmented STAT3 and VDR-related CYP27B1, DEFB4A, and CAMP signaling pathways, MIR337-3p could specifically target/bind to both the *TLR4* and *STAT3* 3′-UTRs to depress the TLR4/MYD88 and STAT3 signals. Data also suggest that MIR337-3p impairing either TLR4/MYD88 or STAT3 signals indeed reduces VDR antimicrobial response involving VDR-related CYP27B1, DEFB4A, and CAMP pathways (**Figure 8F**).

### Innate Human PBMCs With High MIR337-3p Exhibited a Reduced Ability to Mount Fast-Acting Immunity; Mir337-3p Inhibitor Treatment Reversed Mir337-3p-Enhanced Pathogenicity in Mycobacterial Infection of Mice

It is likely that the STAT3 and TLR4/MYD88 signals activate or enhance both the macrophage antimicrobial response and the fast-acting antimycobacterial immunity involving innate-like Vγ2Vδ2 T cells (5), MAIT cells, and NK/ILCs in PBMCs or lymphocytes. We therefore hypothesize that MIR337-3p not only could target/depress VDR antimicrobial response but also suppress fast-acting antimycobacterial immunity, leading to enhanced pathogenicity of infection. To test this hypothesis, we examined whether human PBMCs expressing higher MIR337-3p exhibited a reduced fast-acting innate immunity against mycobacterial growth in cellular models and whether higher expression of miR-337-3p could correlate with greater mycobacterial burdens or pathogenicity.

First, we identified some uninfected subjects who exhibited significantly higher MIR337-3p levels in PBMCs than those in other humans (**Figure 8A**, right panel) when we screened the cohort in the parallel *STAT3* single-nucleotide polymorphism (SNP) study (7). We then examined whether the MIR337-3p-high PBMC samples exhibited a reduced ability to mount fast-acting innate immunity against TB infection when compared with that in the MIR337-3p-low group. For proof of concept, we used the PBMC-based fast-acting innate immunity assays recently published by us and Wang et al. (7). Thus, innate PBMCs were transiently cocultured with BCG-infected A549 cells and tested for the ability to limit/control intracellular mycobacterial infection from infected A549 lung cells. Use of BCG-infected cells, instead of direct BCG exposure to PBMCs, would better control individual variations of BCG uptake. The use of BCG, not *Mtb*, was justified because published studies demonstrated that BCG was similar to *Mtb* in transient short-term (3-day) infection of monocytes/macrophages (6, 22). Such 3-day infection of A549 and monocytes/macrophages (acquired from A549) in cocultures allowed us to evaluate fast-acting innate immunity components including macrophage VDR-antimicrobial activities and γδ T-mediated antimycobacterial immunity in PBMCs. Surprisingly, BCG CFU counts in cocultures from MIR337-3p-high PBMCs were significantly greater than those from MIR337-3p-low controls (**Figure 8A**, left panel). Since the innate PBMCs inhibited mycobacteria in A549 cells and in monocytes/macrophages spread from infected A549 cells, we interpreted growth inhibition as fast-acting innate antimycobacterial immunity as we recently did (7). The results in our fast-acting innate immunity model suggested that the higher MIR337-3p reduced the ability of innate populations in PBMCs to mount fast-acting cellular immunity against intracellular mycobacterial infection (**Figure 8A**, left panel).

Second, we performed a proof-of-concept *in vivo* experiment, in which mice were repeatedly infected with high-dose BCG or PBS control and then assessed for correlation between clinical status (infection pathogenicity), MIR337-3p levels, and CFU counts in lungs at 3 weeks after infection. Surprisingly, a higher expression of MIR337-3p after BCG infection of mice coincided with much greater CFU counts in lungs and even the death of infected animals at ~2 weeks after infection (**Figures 8B, C**). Notably, the expression levels of MIR337-3p in lungs of alive and dead mice were >5 times and 15 times greater, respectively, than those of PBS controls (**Figure 8B**). Moreover, the greatest level of MIR337-3p coincided with unexpected deaths and highest BCG infection burdens, with dead mice showing ~100 times higher BCG CFU counts in lungs than alive animals at their endpoints (**Figure 8C**).

Finally, we sought to determine whether intervention by Mir337-3p inhibitors could reverse Mir337-3p-enhanced pathogenicity of BCG infection in mice. We injected mice with lentivirus expressing inhibitors to knock down Mir337-3p after BCG challenge. At 3 weeks after BCG infection, lung and spleen were homogenized and lysate was assessed for Mir337-3p and diluted/plated for CFU counts. We found that Mir337-3p inhibitor treatment of BCG-infected mice reduced Mir337-3p levels and reversed Mir337-3p-mediated increases in CFU counts in the lung and spleen of the inhibitor-treated mice than those in control animals (**Figures 8D, E** **)**. These results demonstrated that knockdown of Mir337-3p expression could reverse the Mir337-3p-enhanced BCG infection in mice.

Together, mycobacterium-driven MIR337-3p, while depressing both TLR4 and STAT3 signals, reduced the ability of innate cell populations to mount VDR antimicrobial response and fast-acting cellular immunity against mycobacterial infection (**Figure 8F**). These disruptions led to enhanced pathogenicity of bacilli infection.

## Discussion

TB or mycobacterial infection clearly drives increases in MIR337-3p expression. While high-level Mir337-3p/MIR337-3p expression was induced by mycobacterial infection in mice and human macrophages/lung epithelial cells, TB patients showed significantly increased MIR337-3p in CD14^+^ monocytes/macrophages, innate-like Vγ2^+^ T cells, and CD8^+^ lymphocytes containing NK/innate lymphoid cells/MAIT cells. MicroRNA MIR337-3p/Mir337-3p promoted the mycobacterial entry/infection and replication/growth in host target cells: macrophages THP-1/RAW264.7 and lung epithelial cells A549. Such mycobacterium-driven MIR337-3p targeting of both TLR4/MYD88 and STAT3 signals led to depression of VDR antimicrobial CYP27B1/DEFB4A/CAMP and fast-acting innate immune responses.

This single MIR337-3p, characterized by having two specific targeting/binding sites, appears to represent a new or rare finding because one single miRNA usually targets/binds to the 3′-UTR of a single particular gene (not ≥2 binding sites), downregulating the single target gene/protein expression and function (11–13). Our experiments also provided previously unreported data demonstrating that LPS/TLR4/MYD88 stimulation enhanced expressions of the downstream VDR antimicrobial pathway genes of *CYP27B1*, *VDR*, and *DEFB4A*. We also showed that MIR337-3p clearly reduced the ability of LPS/TLR4/MYD88 to augment macrophage expression of CYP27B1, a 25-hydroxy vitamin D3 1-α-hydroxylase for producing antimycobacterial bioactive form of vitamin D [1,25 (OH)2D3] (26). Concurrently, the current study added new information to our previously defined STAT3–VDR axis (7), as we now demonstrated that miR-337-3p indeed decreased STAT3 expression by binding the 3′-UTR of the *STAT3* and thus suppressed the downstream VDR-related antimycobacterial CYP27B1, DEFB4A, and CAMP pathways.

Our cellular models also uncovered a new observation that while activation of TLR4/MYD88 by LPS augmented STAT3 signaling, the MIR337-3p-induced disruptions of TLR4/MYD88 and STAT3 appeared to decrease both VDR-related antimicrobial response in macrophages and fast-acting antimycobacterial immunity components in PBMCs. In fact, the TLR4/MYD88-driven activation of STAT3 is supported by the published data indicating that TLR4 activation induces a signaling cascade recruiting TBK-1 for STAT3 S727 phosphorylation/activation, which facilitates metabolic reprogramming and inflammatory function in macrophages or others (27). STAT3 activation likely enhances activation and immune responses of macrophages and lymphocytes, as STAT3 is an important transcriptional factor involved in a broad spectrum of biological functions including producing anti-TB IL-17 or other cytokines (28). From these standpoints, it is explainable that MIR337-3p impairing of TLR4/MYD88 and STAT3 signals can reduce both the macrophage VDR-related antimicrobial response and the ability of innate lymphocytes to mount fast-acting antimycobacterial immunity, as we see in our cellular models and BCG-infected mice. These help to explain a decreased antimicrobial killing of mycobacteria in macrophages/lung epithelial cells and an increased mycobacterial entry/infection and growth in cells and mice. Our findings are also consistent with the published reports regarding TLR4/MYD88 activation/function. It has been shown that LPS-activated TLR4/MYD88 signal induces a robust pro-inflammatory response (29) and that polymorphisms in *TLR4* indeed could predict both tuberculin skin test (TST) conversion and active TB among contacts of TB patients (30). Furthermore, TLR4 signaling could promote the ability of macrophages to exert multiple anti-*Mtb* immune mechanisms (29).

While downregulation of STAT3 pathway impaired VitD-mediated mycobactericidal activities [**Figure 4** in Wang et al. (7)], it is technically challenging to manipulate the dual knockdowns of both TLR4/MYD88 and the STAT3 pathways vs. the single knockdown of TLR4 alone or STAT3 alone for MIR337-3p dual-targeting effects (because MIR337-3p targets both TLR4 and STAT3 pathways, head-to-head comparisons are required to reach a scientifically sound conclusion). Moreover, because LPS ligand stimulation is required to optimally uncover MIR337-3p downregulation of TLR4/MYD88-driven VitD pathways (**Figure 6**) and because LPS ligand stimulation can increase the ability of macrophages to exert multiple mycobactericidal activities/mechanisms including phagocytosis, ROS production, and destruction of *Mtb* (29), it is difficult to separate the MIR337-3p-LPS-TLR4-VitD-specific control of *Mtb* from multiple/complex LPS-TLR4 mycobactericidal activities. Despite these challenges, we established that MIR337-3p targeted/regulated both the TRL4/MYD88 (**Figure 5**) and the STAT3 (**Figure 7**) pathways in the upstream and then impaired the downstream VitD-driven VDR/CYP21B1/CAMP/DEFB4A antimicrobial signals, with these impairments being reversed by specific inhibition of MIR337-3p but not controls (**Figures 4**, **6**, **7**). Moreover, we demonstrated that MIR337-3p regulation could reduce anti-*Mtb* and anti-BCG immunity (**Figure 3**), and we particularly showed that MIR337-3p indeed downregulated the upstream STAT3 and the downstream VDR/CYP27B1/CAMP/DEFB4A to reduce VitD-mediated control of mycobacterial infection (**Figures 4**, **7**). This was consistent with the observation that silencing STAT3 impaired VDR-mediated mycobactericidal activities (7). Concurrently, MIR337-3p impairing of the upstream LPS-activated TLR4/MYD88 could similarly downregulate the downstream VDR/CYP21B1/CAMP/DEFB4A (**Figures 5**, **6**), which might also impair VitD-activated VDR signals and VDR-mediated mycobactericidal activities, as shown in **Figure 4**.

The current study provides new and innovative data supporting the hypothesis that TB-driven single MIR337-3p species could specifically target/impair both TLR4/MYD88 and STAT3 activation signals, inhibiting VDR-activated antimicrobial response and fast-acting anti-TB immunity, leading to enhanced pathogenicity (**Figure 8F**). Our data provide a principle relevant to initial or early human TB infection, since we address the possibility that mycobacterium-driven MIR337-3p can enhance initial pathogenicity by targeting TLR4/MYD88 and STAT3 to impair the capabilities of VDR antimicrobial response and fast-acting antimycobacterial immunity. The use of acutely BCG-infected cellular and mouse models is justified, as we assess mycobacterial entry/replication/infection in short-term 4-h or 3-day cells and 3-week mice rather than address TB pathology, disease, or evolution of host–*Mtb* interaction in chronic setting. Notably, despite using BCG (not *Mtb*), our short-term cellular/mouse infection models clearly demonstrated that MIR337-3p reduced macrophage VDR antimicrobial response, leading to enhanced BCG entry/replication/infection in cells as well as the ability of innate cell populations to mount fast-acting immunity. Moreover, a higher expression of Mir337-3p after BCG infection of mice coincided with much greater CFU counts and even death, with Mir337-3p inhibitor treatment reversing Mir337-3p-enhanced pathogenicity. It is likely that MIR337-3p downregulation of TLR4/STAT3 also reduces the ability of macrophages to exert the bactericidal phagosome–lysosome fusion. It is also possible that impairing of VDR antimicrobial and fast-acting innate immune responses would compromise the development of adaptive immune response, leading to an enhanced mycobacterial infection or progression to TB. These notions help to explain why low-pathogenic BCG infection was able to induce death in some mice by driving remarkably high Mir337-3p expression and extremely great BCG CFU counts (8). Thus, the findings in the current study raise the possibility that definitive clinical studies can be conducted to confirm the concept that TB bacilli induce or enhance TB pathogenicity/disease through driving miRNA upregulation for impairing VDR antimicrobial response and fast-acting innate immunity or other pathways.

## Data Availability Statement

The original contributions presented in the study are included in the article/**Supplementary Material**. Further inquiries can be directed to the corresponding authors.

## Ethics Statement

The studies involving human participants were reviewed and approved by the ethics committee of Shanghai Pulmonary Hospital. The patients/participants provided their written informed consent to participate in this study. The animal study was reviewed and approved by the ethics committee of Shanghai Pulmonary Hospital.

## Author Contributions

HS designed the experiments and wrote the article. All authors contributed to the article and approved the submitted version.

## Funding

This work was supported by the Chinese National Major Projects Grants (2018ZX10731301-006-001 to HS), Shanghai Science and Technology Committee Basic Research Grant (No. 20JC1417800 to HS, 20dz2210404 and DGF501022/028/002 and 20ZR1406200 to FW, 2019LJ13 to WS), and National Natural Science Foundation of China Grants (81401711 to FW, 31970876 and 32070943 to HS).

## Conflict of Interest

The authors declare that the research was conducted in the absence of any commercial or financial relationships that could be construed as a potential conflict of interest.

## Publisher’s Note

All claims expressed in this article are solely those of the authors and do not necessarily represent those of their affiliated organizations, or those of the publisher, the editors and the reviewers. Any product that may be evaluated in this article, or claim that may be made by its manufacturer, is not guaranteed or endorsed by the publisher.

## Supplementary Material

Supplementary Figure 1 The Western blot figures with molecular markers (PageRuler™ Prestained Protein Ladder, 10–180 kDa, Thermo Scientific, Cat: 26616) were shown. Molecular markers with the individual target proteins corresponded to them in SDS-PAGE. **(A)** showed repeat of TLR4 (Abcam, Cat: ab13556) expression in **Figure 5B**. **(B)** showed repeat of MYD88 (Cell Signaling Technology, Cat: 4283S) expression in **Figure 5F**. **(C)** showed repeat of STAT3 (Cell Signaling Technology, Cat: 9139S) expression in **Figure 7D**.
