# Supplementary figures and images for: MIR337-3p Enhances Mycobacterial Pathogenicity Involving TLR4/MYD88 and STAT3 Signals, Impairing VDR Antimicrobial Response and Fast-Acting Immunity
Source: Front Immunol. 2021 Nov 29;12:739219. doi: 10.3389/fimmu.2021.739219 (PMC8666424; doi:10.3389/fimmu.2021.739219)

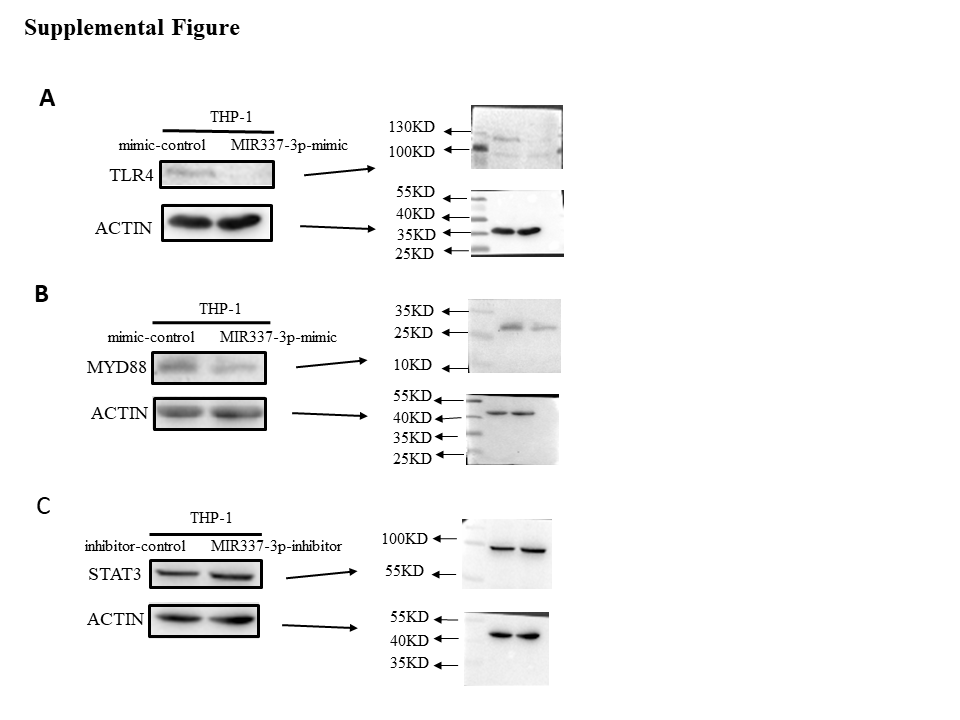

Supplement: Supplementary Figure 1 — The Western blot figures with molecular markers (PageRuler™ Prestained Protein Ladder, 10–180 kDa, Thermo Scientific, Cat: 26616) were shown. Molecular markers with the individual target proteins corresponded to them in SDS-PAGE. (A) showed repeat of TLR4 (Abcam, Cat: ab13556) expression in Figure 5B . (B) showed repeat of MYD88 (Cell Signaling Technology, Cat: 4283S) expression in Figure 5F . (C) showed repeat of STAT3 (Cell Signaling Technology, Cat: 9139S) expression in Figure 7D . [file Image_1.tif]
